# Taenia saginata infection: A rare case of jejunal perforations from Ethiopia: A case report

**DOI:** 10.1016/j.ijscr.2025.110974

**Published:** 2025-01-27

**Authors:** Lohide Daniel Lopura, Engida Abebe Gelan, Daniel Getaw Mengiste, Solomon Fekadu Yehualawork

**Affiliations:** a Department of Surgery, St. Paul's Hospital Millennium Medical College (SPHMMC), P.O.BOX 1271, Addis Ababa, Ethiopia; b Department of Surgery, Endocrine-Breast and Transplant Surgeon, St. Paul's Hospital Millennium Medical College (SPHMMC), P.O.BOX 1271, Addis Ababa, Ethiopia

**Keywords:** Jejunal perforations, Taenia saginata, Case report, Ethiopia

## Abstract

**Introduction and importance:**

T. saginata, a global parasite, is still a considerable burden in low-income countries. It is transmitted to humans through the consumption of undercooked/raw beef. Although taeniasis can cause an acute abdomen, taeniasis-induced bowel perforation is rarely encountered, and only a few cases have been reported.

**Methods:**

This work has been reported in line with the SCARE criteria.

**Case presentation:**

A 44-year-old male experienced abdominal pain, vomiting, anorexia, and fever over 24 h. He had a 5-month history of mild gastrointestinal symptoms. His clinical picture and imaging findings suspected a viscus perforation. An exploratory laparotomy revealed jejunal perforations with an adult Taenia tapeworm. Postoperatively, significant events occurred.

**Clinical discussion:**

T. saginata is a common infection in regions with high undercooked/raw beef consumption. In Ethiopia, raw beef consumption is common and is the most likely source of infection in this case. T. saginata infection can cause acute abdomen with intestinal obstruction, but rarely does it induce intestinal perforations. Only a few taeniasis-related bowel perforations necessitating surgical intervention have been reported from Lebanon, Nepal, and northern Iran. This patient may be the only reported case of teniasis-related bowel perforations complication with postoperative fatality, with other cases having uneventful postoperative periods. The cause of taeniasis-related intestinal perforation remains unclear. Bedside qSOFA scoring system can predict death risk in sepsis patients. In septic shock, rapid resuscitation is crucial.

**Conclusion:**

The case report emphasizes the importance of Taenia saginata as a differential diagnosis for intestinal perforation, particularly in endemic regions, and suggests clinical suspicion of taeniasis as a possible cause. Rapid resuscitation and source control are crucial for sepsis patients. The qSOFA scoring system can predict death risk, with a 3 score indicating higher mortality.

## Highlights

• T. saginata, a global parasite, is prevalent in low-income countries.
• It is rare for taeniasis to cause bowel perforation requiring surgical intervention.
• The most common reported surgical complication of taeniasis is mechanical bowel obstruction.
• In endemic areas, taeniasis-related acute surgical abdomen may be a probable etiology.
• Clinical suspicion of taenia-related acute abdomen should be considered in endemic areas.

## 1 Introduction

Taenia saginata is a prevalent zoonotic tapeworm infection, with an estimated global prevalence of 60–70 million carriers [2]. Its transmitted to humans through undercooked meat from cattle, which is the intermediate host of the parasite's infective larval stage [3]. Infected individuals are often asymptomatic for years, and may exhibit impulsive transit of proglottids [4]. Vague symptoms of nausea, vomiting, abdominal discomfort, increased hunger, weight loss, or pruritus ani may be present. The diagnosis is typically confirmed by history, stool samples, and Taenia species examination [5]. T. saginata infection can cause an acute abdomen with intestinal obstruction, but it rarely presents with intestinal perforations.

We report a rare case of jejunal perforations likely induced by T. saginata infection at an academic hospital and review similar cases. This work has been reported in line with the SCARE criteria [1].

## 2 Case presentation

A 44-year-old male, a casual laborer, presented to the emergency department with a complaint of worsening of abdominal pain over a 24-hour duration. The pain began as central abdominal pain, then gradually became generalized and was associated with abdominal distention, multiple episodes of bilious vomiting, anorexia, and fever. Prior to the patient's acute presentation, he had a 5-month history of self-limiting intermittent crampy abdominal pain, dyspepsia episodes, occasional constipation, loose motions, and slight weight loss of the same duration. He is a regular smoker of 20 pack years and an occasional alcohol drinker. He had no medical or surgical history. No family history of a specific chronic disease.

**Fig. 1:**
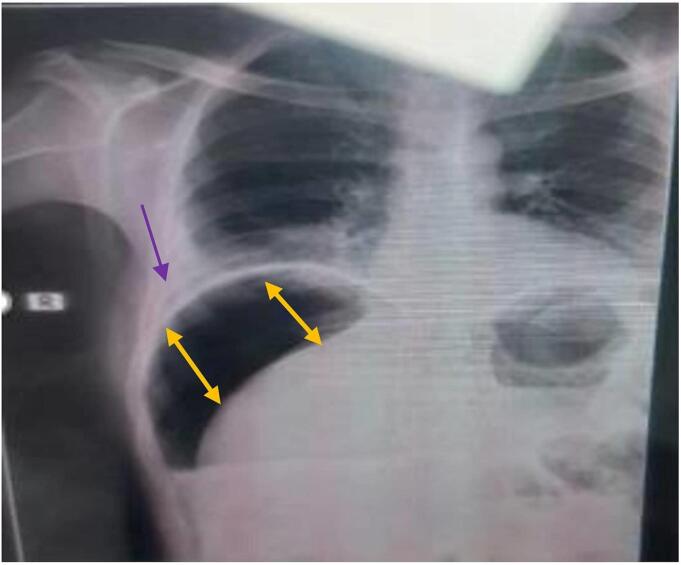
Chest X-ray at hospital admission: Shows free air under the diaphragm (double yellow arrows), obliterated right costo-diaphragmatic angle (purple arrow). (For interpretation of the references to colour in this figure legend, the reader is referred to the web version of this article.)

At admission, the patient looked sick, hypotonic, and confused with a GCS of 14/15. He had dry mucous membranes and pink conjunctiva. Febrile to touch with an axillary temperature of 38.5°C, tachycardia with a feeble pulse of 140/min, hypotension with BP of 70/40 mmHg (MAP = 50 mmHg), tachypnea with a respiratory rate of 25 breaths per minute, and SaO2 of 94% on room air. Abdominal examination revealed diffusely tender abdomen, involuntary guarding and rebound, diminished bowel sounds, and formed loose stool on digital per rectal examination. An erect chest X-ray revealed pneumoperitoneum, and right basal Lung opacities with loss of costo-diaphragmatic angle on the right side, likely right minimal pleural effusion (refer to Fig. 1 *below*). Laboratory parameters showed; WBC 4400/mm3 with left shift of 91% neutrophils, 72% lymphocytes, Hg 18g/dL, and platelets of 319,000/uL. Blood chemistry showed; creatinine 1.96 mg/dL, urea 59.3 mg/dL, Na+ 130 mmol/dL, K+ 4.4 mmol/dL, Cl- 93.2 mmol/dL.

**Fig. 2:**
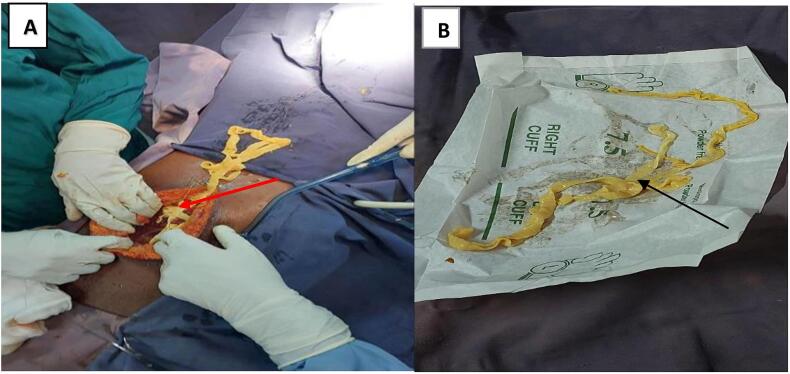
(A) T. saginata free in peritoneum(red arrow) and (B) removed T. saginata worm laid on surgical glove(black arrow), upstretched it measured ∼3 m. (For interpretation of the references to colour in this figure legend, the reader is referred to the web version of this article.)

**Fig. 3:**
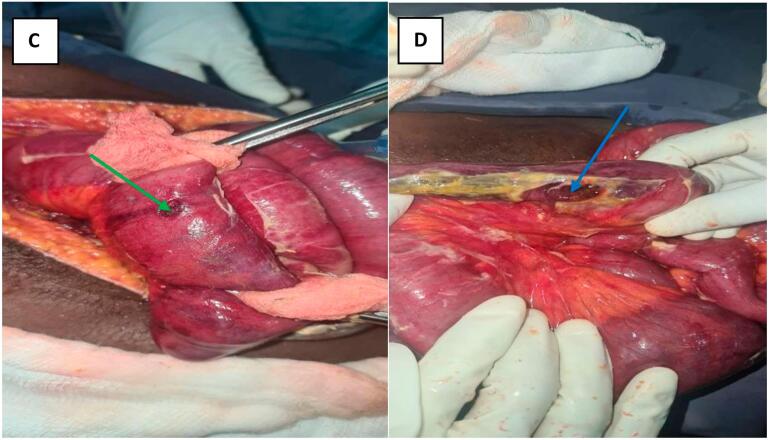
(C) First site (green arrow) of jejunal perforation and (D) second site (blue arrow) of jejunal perforation after removal of T. saginata from peritoneum. (For interpretation of the references to colour in this figure legend, the reader is referred to the web version of this article.)

Thus with a presumed diagnosis of generalized peritonitis due to a perforated peptic ulcer plus septic shock of Gastrointestinal focus, AKI due to pre-renal azotemia and mild hyponatremia. Resuscitation immediately started after securing 2-large cannula intravenous line. Starting with fluid bolus; 30 mL/kg of normal saline, then followed by maintenance fluid 25 mL/kg/24 h given in hourly amount, second fluid bolus repeated with no much increment in BP records. Vasopressor initiated; Norepinephrine 0.1 μg/kg/min infused with maintenance fluid. Empirical antibiotic given; intravenous ceftriaxone 50 mg/kg/day in divided dose twice daily, intravenous metronidazole 15 mg/kg/day in divided dose three times daily, and intravenous paracetamol 15 mg/kg/day thrice per day. The Resuscitation time took 3 h with the patient initially responding to resuscitation; pulse rate decreased to 125/min from 140/min on arrival, BP increased to 100/60 mmHg (MAP=73 mmHg) from initial 70/40 mmHg (MAP=50 mmHg), he produced 200 mL of Urine output (∼1 mL/kg/min) collected in the urine bag over 3 h time from initial anuric state. However, the blood pressure remained in borderline range and patient could not sustain normotensive state without need of vasopressor. He was taken to surgery while receiving low dose vasopressor infusion. The central venous pressure measurement was not possible due to hospital resource limitations. While in emergency room used the quick Sepsis-Related Organ Failure Assessment (qSOFA) score to assess the patient's organ function and predict the prognosis. The patient had a score of 3(*GCS 14/15, RR 25, systolic BP 100 mmHg*) which predicted a higher risk of death of 3–14 times the rate of in-hospital mortality. We Subsequent exploratory laparotomy decided with ICU bed prepared in advance for postoperative nursing and mechanical ventilator support. Laparotomy revealed a 3 m long tapeworm freely movable in the peritoneal cavity (refer to Fig. 2; *labelled A and B below*), 3 L of yellow-green fluid, fibrin pseudo-membrane deposits, and two mid-jejunal perforations located at the ante-mesenteric border ∼20 cm distal from the ligament of Treitz, each 5 cm apart, equal in size of ∼1 cm × 1 cm (Refer to Fig. 3; *Labelled C and D below*).

The patient underwent primary closure of perforation sites after removal the Taenia worm and cleansing the peritoneal cavity. The primary repair was chosen to control intestinal contents spillage and sepsis source. Resection and stoma creation could have been an option, but the unstable patient's shock state with increased vasopressor doses necessitated shortening the duration of surgery and ICU transfer for optimization, and a second look in 24–48 h was considered. Thus a damage control approach was the preferable in this case. Postoperatively the patient was transferred intubated from operation theatre directly to ICU; the patient was put on Mechanical Ventilator support VAC mode, Fi02 100% and continuously required double Vasopressors with addition of Dopamine 5 μg/kg/min infusion. The patient died 8 h post-operatively in ICU with possible cause of death being multi-organ failure secondary to refractory septic shock. The patient's deterioration may be due to significant peritoneal contamination from double perforation sites, resulting in a significant spill of septic intestinal fluid, and a late presentation to the emergency room after 24 h of worsening abdominal symptoms. The patient denied any history of prior passage of proglottids; however, based on microscopic analysis of the morphology and uterus branches, T. saginata was suggested as the parasite. Due to hospital resource limitations, PCR tests are not possible. Histopathological examination reveals granulation tissue and inflammation.

## 3 Discussion

T. saginata is prevalent globally, especially in regions with high consumption of undercooked beef [6]. The incidence of T. saginata in cattle production in areas of eastern and southern Africa is still unclear [7]. In Ethiopia there have been a high percentage of tapeworm self-reporting (45.0–64.2%) [8]. Thus suggesting that raw beef consumption is the most likely source of infection in this case. Taeniasis-related bowel perforation requiring surgical intervention is rare. The most common complication is mechanical intestinal obstruction caused by contact with Taenia bolus-enhancing bowel mucosa.

Very limited cases of Taeniasis-related bowel perforation have been stated. One of the few of such rare case was a 69-year-old Lebanese male with abdominal pain, nausea, and vomiting was diagnosed with preoperative impression of a perforated duodenal ulcer; however an urgent laparotomy revealed a yellow-green secretion, fibrin pseudo-membrane deposits, a perforation in the proximal jejunum and a swinging Taenia saginata [9]. This is comparable to our intra-operative findings which revealed an extra-luminal tapeworm, a 2-site mid jejunal perforations ∼20 cm distal from ligament of treitz at the ante-mesenteric border. Also in 2022, a 50-year-old Nepali male with abdominal pain and vomiting was diagnosed with viscus perforation, and an adult tapeworm was surgically removed during exploratory laparotomy [5]. Furthermore in Northern Iran, a case of small intestine obstruction, volvulus, and necrosis leading to bowel perforation due to an impacted tapeworm [10]. The patient's intestinal perforations are likely due to taeniasis infection. The extensive ischemic patches and fibrinous adhesions are a result of severe antigenic inflammatory reaction on the intestinal and abdominal wall. Moreover, given the patient's history of intermittent vague abdominal symptoms for 5 months, histopathological examination indicated granulation tissue and inflammation; this makes other differential diagnoses less likely. The exact cause of taeniasis-related intestinal perforation is unclear, but it could be due to local irritative reactions or the direct effect of Taenia [11]. Since this patient presented in shock, rapid resuscitation and source control are crucial in patients with profound sepsis, and to anticipate a poorer prognosis, the qSOFA scoring system done at bedside can easily be used to assess organ function and predict death risk. The patient had a qSOFA score of 3, which predicted a higher risk of death of 3–14 times the rate of in-hospital mortality [12]. The patient's deterioration may be due to double perforation sites, causing significant septic fluid spill, and late emergency room presentation, unlike similar case reports with single perforation sites, which could have offered reduced peritoneal contamination and uneventful postoperative outcomes with no recurrences. This patient may be a case of teaniasis-related bowel perforation complication with postoperative fatality.

## 4 Conclusion

The case report highlights that Taenia saginata, a worm of about 3 m or more long in length, should be on the list of differential diagnoses of intestinal perforation in any site of the gastrointestinal tract, especially in an endemic region. Clinicians should maintain a clinical suspicion of taeniasis as a possible cause. Rapid resuscitation and source control are important factors to save patients with sepsis and to anticipate a poorer prognosis; the qSOFA scoring system predicts death risk, with a score of 3 indicating higher mortality.

## Author contribution

Lohide Daniel Lopura, M.D -Primary operating surgeon, Study concept and plan, writing the paper and literature review.

Engida Abebe Gelan, M.D, Editing and critical Review of the article.

Daniel Getaw Mengiste, M.D -Editing the manuscript, patient management, data collection, and critical review.

Solomon Fekadu Yehualawork, M.D - Editing the manuscript, patient management, data collection, and critical review.

## Consent

Written informed consent was obtained from the patient mother who is the primary care giver, in their native language, for publication of non-identifying information including accompanying intraoperative images. A copy of the written consent is available for review by the Editor-in-Chief of this journal on request.

## Ethical approval

Ethical approval is deemed unnecessary by the ethical committee, as this is a single case encountered during practice, and it doesn't involve human or animal experiments. This particular study is waived from ethical approval by the St. Paul's Hospital Millennium Medical College Institutional Review Board.

## Guarantor

Lohide Daniel Lopura, MD.

## Research registration number

N/A.

## Funding

No funding was provided for this case report.

## Conflict of interest statement

No conflict of interest among the authors.

## References

[1] Sohrabi C., Mathew G., Maria N., Kerwan A., Franchi T., Agha R.A. (May 1 2023). The SCARE 2023 guideline: updating consensus Surgical CAse REport (SCARE) guidelines. Int. J. Surg. [Internet].

[2] Dbouk ABCDEFG S, Bazzi ABCDEFG N, Mcheimeche DEF H, Rida Farhat BCD M, Alameh ABCDEFG A, Rakka Corresponding Author M, et al. A 27-year-old Lebanese man with stomach perforation and regurgitation of a beef tapeworm (Taenia saginata): a case report and review of the literature. 2021 [cited 2024 Apr 21]; Available from: https://www.amjcaserep.com/abstract/index/idArt/928355. 10.12659/AJCR.928355 PMC8130976 33980806

[3] Taeniasis/cysticercosis [Internet]. https://www.who.int/news-room/fact-sheets/detail/taeniasis-cysticercosis.

[4] Nematihonar B., Pedram S., Hosseini K., Toutounchi A.H. (2023). Taenia saginata, the incidental find in case of intestinal perforation after blunt trauma and literature review. Int. J. Surg. Case Rep. [Internet].

[5] Bhandari R., Chamlagain R., Sutanto E., Adam H., Dhungana A., Ali A.A. (2022). Intestinal perforation due to adult tapeworm of taenia: a case report and review of the literature. Clin. Med. Insights Case Rep..

[6] Bachar A., Elabbassi T., Abdoulaye H.B., Lefriyekh M.R. (2020). Taenia saginata: the solitary enemy. Asian J. Case Rep. Surg. [Internet].

[7] Dermauw V, Dorny P, Braae UC, Devleesschauwer B, Robertson LJ, Saratsis A, et al. Epidemiology of Taenia saginata taeniosis/cysticercosis: a systematic review of the distribution in southern and eastern Africa. [cited 2024 Apr 21]; Available from: 10.1186/s13071-018-3163-3. PMC6219070 30400948

[8] Jorga E., Van Damme I., Mideksa B., Gabriël S. (2020). Identification of risk areas and practices for Taenia saginata taeniosis/cysticercosis in Ethiopia: a systematic review and meta-analysis. Parasites and Vectors [Internet].

[9] Bekraki A., Hanna K. (2016). Peritonitis caused by jejunal perforation with Taenia saginata: report of a case. J. Parasit. Dis. [Internet].

[10] Soosaraie M., Alizadeh S., Fakhar M. (2010). PP-203 Taenia saginata infection: a rare case of intestinal perforation from northern Iran. Int J Infect Dis [Internet]..

[11] Demiriz M., Gunhan O., Celasun B., Aydin E., Finci R. (Jan 1 1995). Colonic perforation caused by taeniasis. Trop. Geogr. Med. [Internet].

[12] Shahsavarinia K., Moharramzadeh P., Arvanagi R.J., Mahmoodpoor A. (2020). Qsofa score for prediction of sepsis outcome in emergency department. Pakistan J. Med. Sci..

